# CHARACTERISTICS AND LIVING ARRANGEMENTS OF CHILDLESS ELDERLY AMERICANS

**DOI:** 10.1093/geroni/igz038.949

**Published:** 2019-11-08

**Authors:** Xiao Xu, Jersey Liang, BoRin Kim, Mary Beth Ofstedal, James Raymo

**Affiliations:** 1 Yale University, New Haven, Connecticut, United States; 2 University of Michigan, Ann Arbor, Michigan, United States; 3 University of New Hampshire, Durham, New Hampshire, United States; 4 University of Wisconsin-Madison, Madison, Wisconsin, United States

## Abstract

Despite a growing number of Americans without children, information on characteristics of childless older adults and their living arrangements is sparse and often outdated. To address this knowledge gap, we examined data from the 2014 Health and Retirement Study (HRS) on childless Americans age ≥65 years (N=760) and compared with childless elders in 2004 HRS (N=830). All analyses accounted for complex sample design of HRS to generate nationally representative estimates. The proportion of elderly Americans without any living biological or step children increased from 8.1% in 2004 to 9.4% in 2014. Compared to childless elders in 2004, those in 2014 were younger (mean age=76.8 years versus 73.8 years, p<0.01) with a higher proportion completing college education (20.7% versus 37.5%, p<0.01) or were cognitively intact (64.6% versus 73.6%, p<0.01). However, childless elders in 2014 had more chronic conditions than those in 2004 (mean=2.5 versus 2.3, p<0.01). The proportion of childless elders living independently (alone or with a spouse) (81.6% in 2004 versus 82.6% in 2014) or living with others (10.0% in 2004 versus 13.5% in 2014) remained relatively stable, whereas the proportion living in nursing homes declined significantly from 8.4% in 2004 to 4.0% in 2014 (adjusted odds ratio [OR]=0.07, p<0.01). A sensitivity analysis limited to childless elders without biological children showed that they accounted for 9.9% of the elderly population in 2004 versus 11.9% in 2014, while other results were generally similar. These findings can help inform long term care needs of childless elders.

